# Supplementary material for: Deep neural operator-driven real-time inference to enable digital twin solutions for nuclear energy systems
Source: Sci Rep. 2024 Jan 24;14:2101. doi: 10.1038/s41598-024-51984-x (PMC10808608; doi:10.1038/s41598-024-51984-x)
Supplement: Supplementary file 1 — Supplementary Information. [file 41598_2024_51984_MOESM1_ESM.pdf]

Supplementary Material A. PHITS Input

Supplementary Material A.1. Material Composition

The compositions of air and concrete employed in this study are presented in Tables A.1 and A.2. In PHITS, materials can be defined using either atomic ratio or mass ratio. The identification of nuclei can be expressed in terms of the number of protons Z and atomic number A using the following formula:

$$\text{Name of Nuclide} = Z \times 1000 + A. \tag{A.1}$$

Nuclide	Composition[1]
7014	78.08
8016	20.95
Density (g/cm^3)	1.21×10^{-3}

[1] atomic ratio of air

Table A.1. Composition of air

Nuclide	Composition[2]
1001	0.011
11023	0.043
13027	0.145
16032	0.002
20040	0.197
26056	0.085
8016	1.05
12024	0.034
14028	0.592
19039	0.035
22048	0.006
Density (g/cm^3)	2.2

[2] mass ratio of concrete

Table A.2. Composition of concrete

Supplementary Material B. Overall performance of the DeepONet models

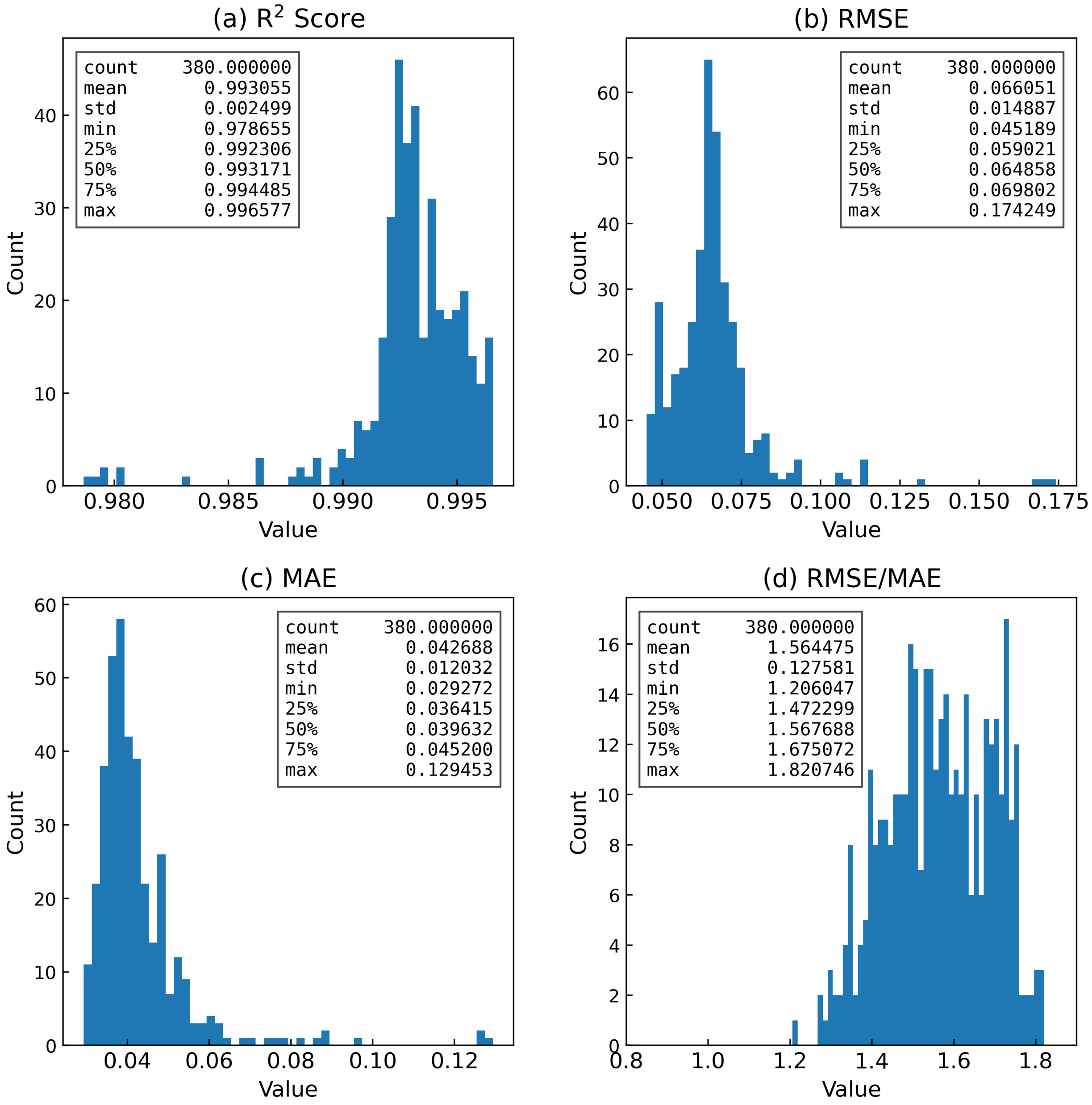

Fig. B.1. DeepONet model trained with the training data Set1. Each pane represents; (a) R^2 score, (b) RMSE, (c) MAE, and (d) RMSE/MAE.

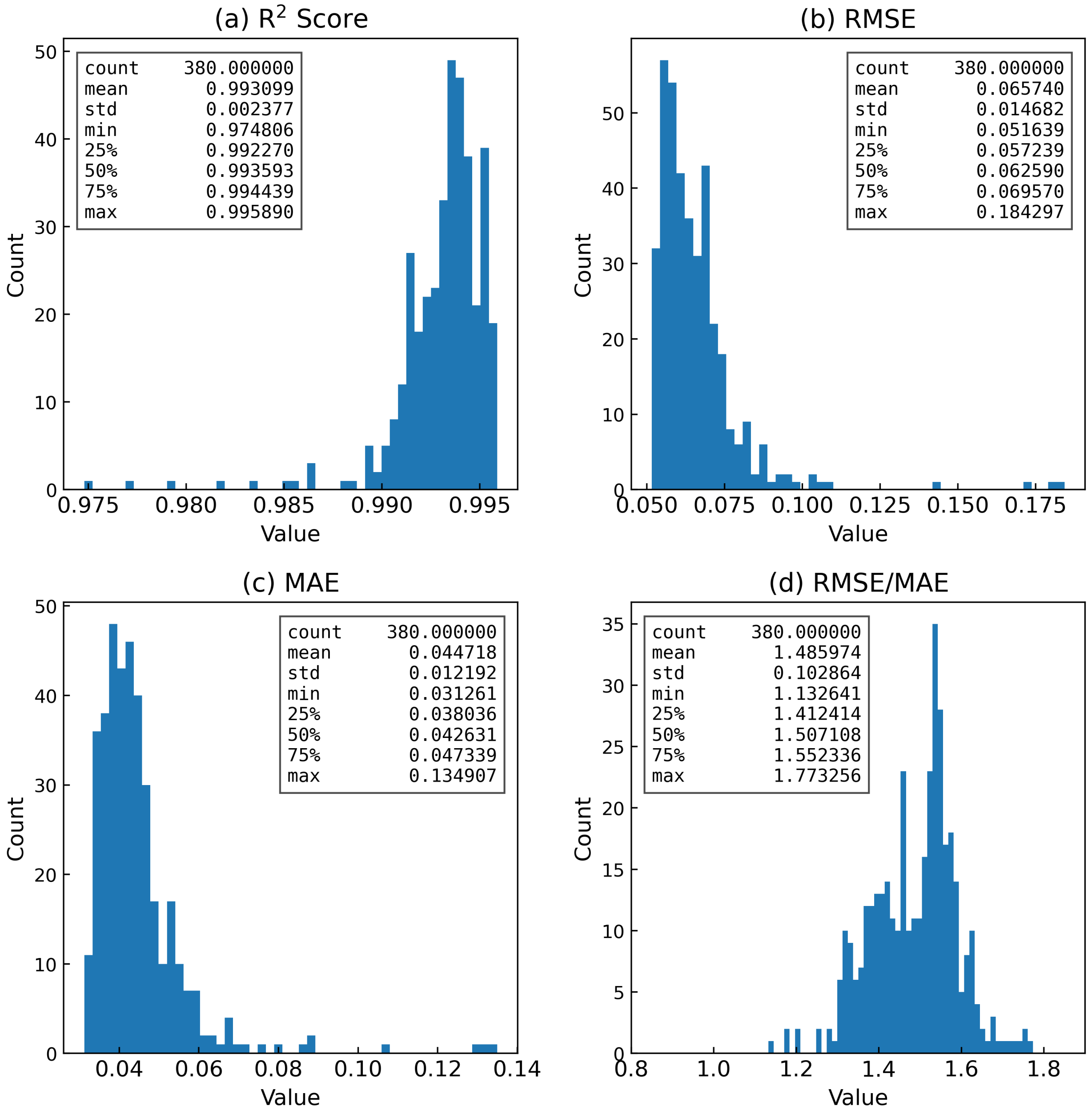

Fig. B.2. DeepONet model trained with the training data Set2. Each pane represents; (a) R^2 score, (b) RMSE, (c) MAE, and (d) RMSE/MAE.

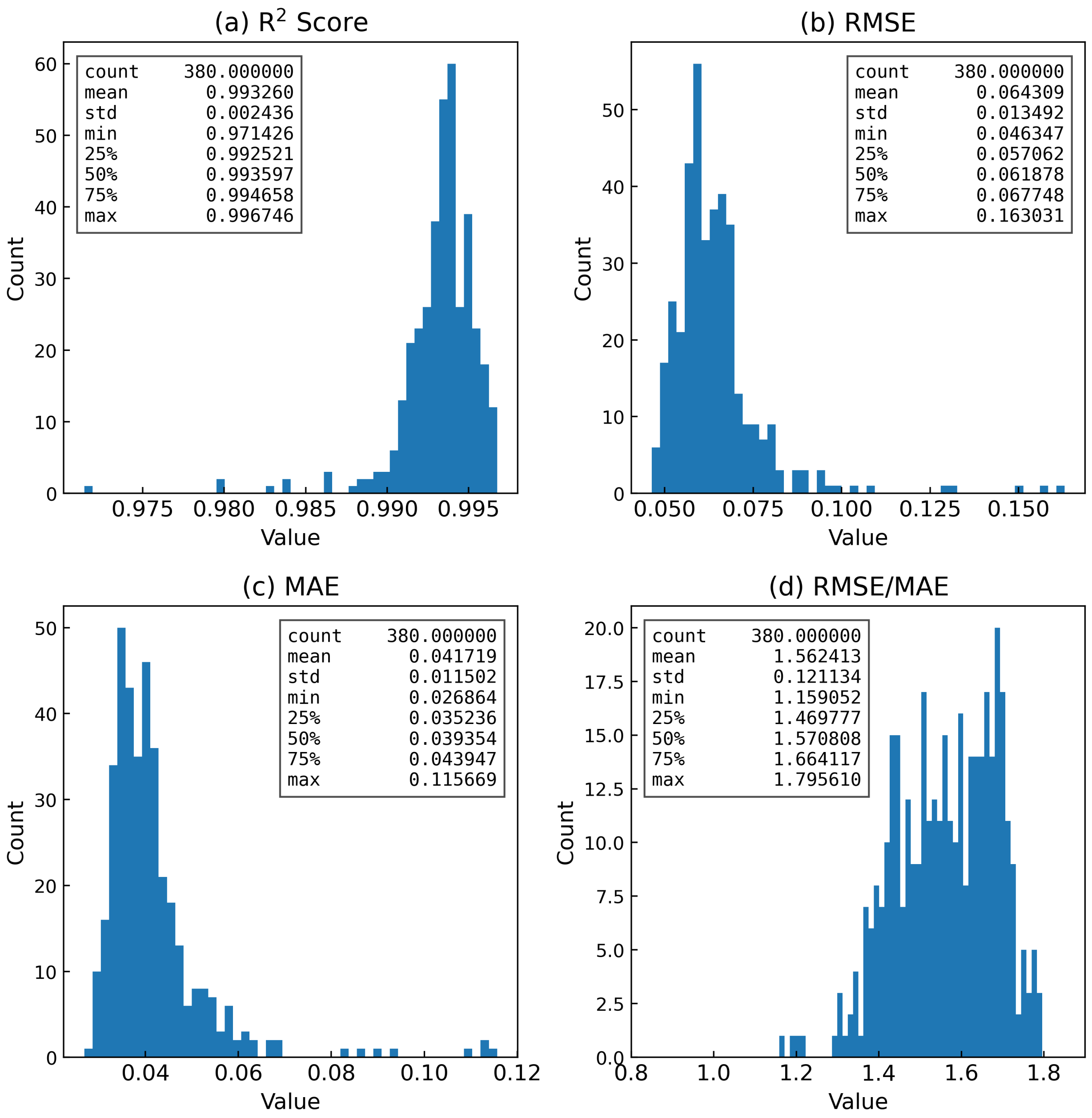

Fig. B.3. DeepONet model trained with the training data Set3. Each pane represents; (a) R^2 score, (b) RMSE, (c) MAE, and (d) RMSE/MAE.

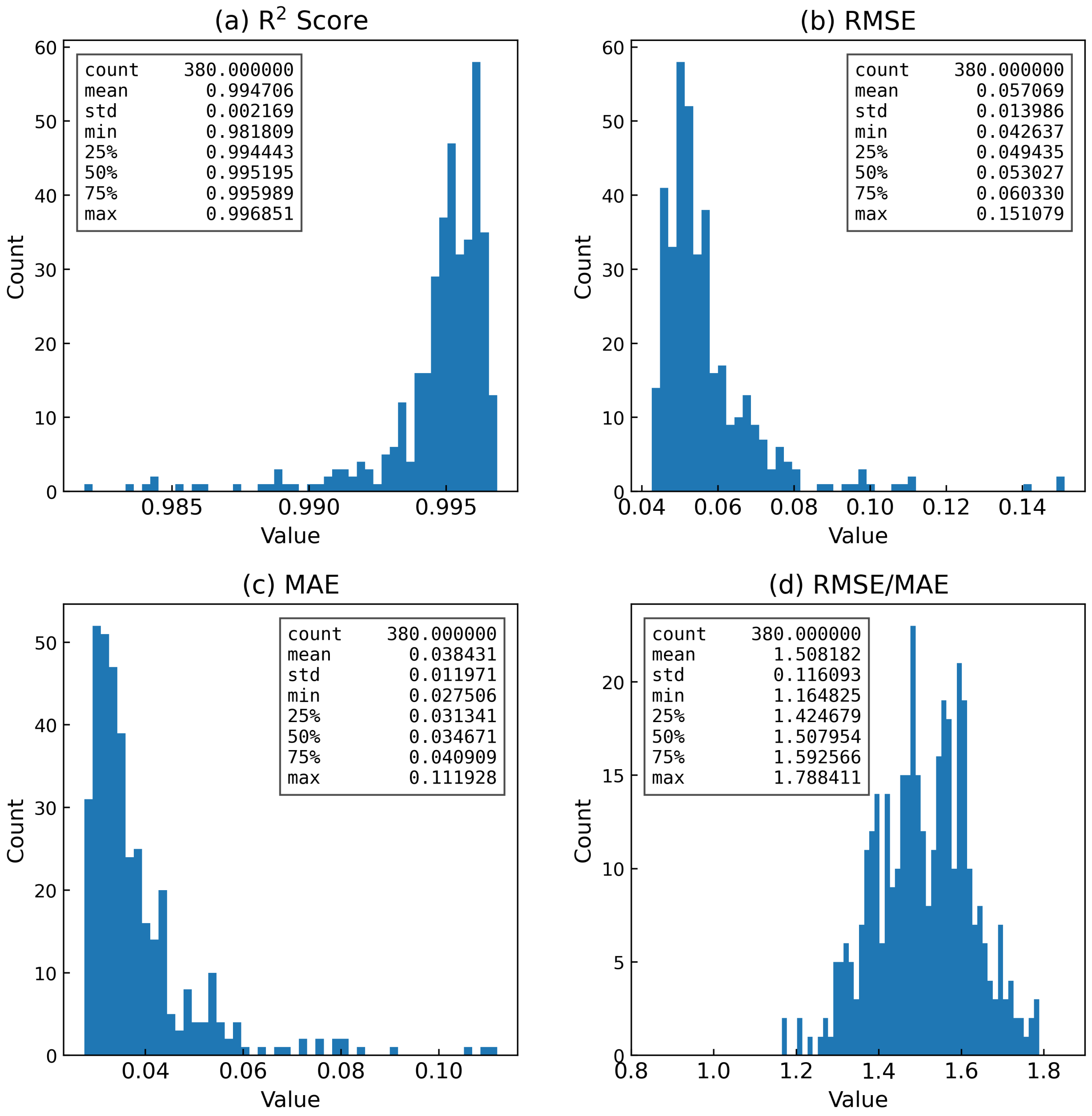

Fig. B.4. DeepONet model trained with the training data Set4. Each pane represents; (a) R^2 score, (b) RMSE, (c) MAE, and (d) RMSE/MAE.

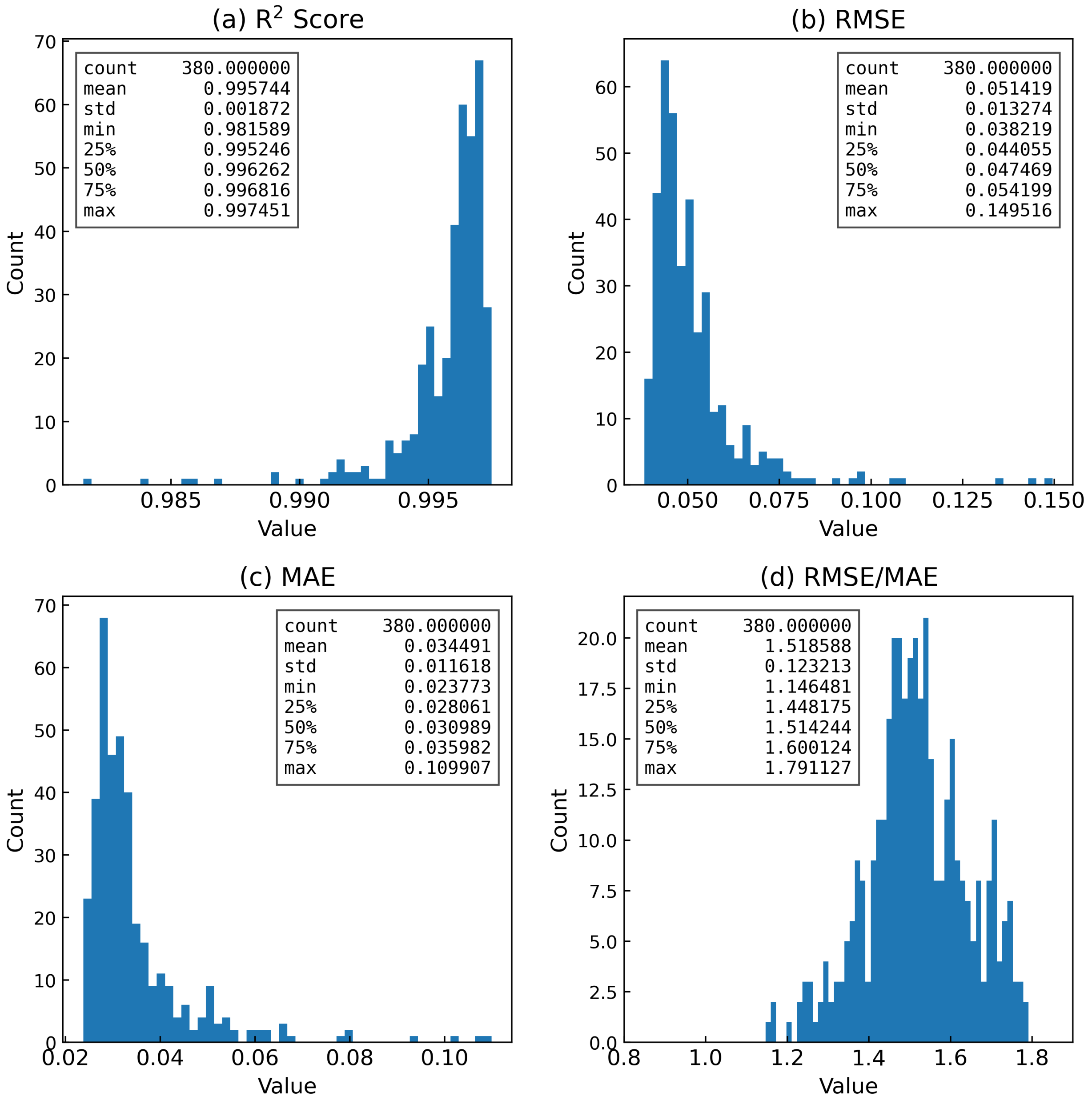

Fig. B.5. DeepONet model trained with the training data Set5. Each pane represents; (a) R^2 score, (b) RMSE, (c) MAE, and (d) RMSE/MAE.

Supplementary Material C. Network Architectures of FCN and CNN

In this study, a comprehensive comparison was conducted between the DeepONet and two conventional neural network methods: Fully-Connected Neural Networks (FCN) and Convolutional Neural Networks (CNN). These networks were implemented using the PyTorch 2.0 deep learning framework, ensuring a consistent and efficient modeling process.

Supplementary Material C.1. FCN

The FCN architectures were designed with simplicity and efficiency in mind, comprising:

- **Input Layer**: With 2 neurons, aligning with the dimensionality of our input data.
- **Hidden Layers**: Multiple architectures were tested, including [2, 512, 512, 1], [2, 1024, 1024, 1], and [2, 2048, 2048, 1], to evaluate different levels of network complexity.
- **Output Layer**: A single neuron, suitable for the regression tasks.
- **Activation Functions**: ReLU, chosen for its effectiveness in mitigating the vanishing gradient problem and facilitating faster training.

Hyperparameter tuning was performed via a random search approach, exploring:

- **Learning Rate**: Ranging from 0.01 to 0.0001.
- **Batch Size**: 32, 64, and 128, to understand the impact on learning dynamics.
- **Number of Epochs**: Fixed at 1024.

The Adam optimizer and Mean Squared Error (MSE) loss function were used. An early stopping mechanism based on validation loss was implemented for enhanced model generalization. The optimal FCN configuration was identified through validation loss minimization, as summarized in Table C.3.

Hyperparameter	Value
Number of Hidden Layers	[2, 1024, 1024, 1]
Learning Rate	0.01
Batch Size	64
Number of Epochs	1024

Table C.3. Best Hyperparameters for the FCN Model

Supplementary Material C.2. CNN

For the CNN models, the training data was reshaped to suit the 1D CNN architecture, resulting in an input shape of [3200, 1, 2], where 3200 is the number of samples, 1 is the number of input channels, and 2 is the number of features per sample.

The CNN architecture comprised:

- **Input Layer**: Adjusted to accommodate the reshaped data.

- **Convolutional Layer**: With 1 input channel and varying output channels (1024, 2048, 4096), and a kernel size of 1, to capture features effectively.
- **Linear Layers**: Two subsequent layers leading to the output.
- **Activation Functions**: Tanh, for non-linear transformation.
- **Dropout**: Set at 0.05 to prevent overfitting.

Hyperparameter tuning was conducted similarly to FCN:

- **Output Channels**: 1024, 2048, and 4096 were tested.
- **Kernel Size**: Fixed at 1.
- **Learning Rate**, **Batch Size**, and **Number of Epochs**: Same ranges as FCN.

The training also used the Adam optimizer and MSE, with early stopping based on validation loss. Table C.4 details the best-performing CNN configuration.

Hyperparameter	Value
Output Channels	2048
Learning Rate	0.001
Batch Size	32
Number of Epochs	1000

Table C.4. Best Hyperparameters for the CNN Model

The architecture and hyperparameter tuning of the CNN models were designed to explore the efficacy of convolutional layers in capturing spatial and temporal features in the data. The optimal configuration determined through this process provided a robust basis for comparing the performance of CNNs against the DeepONet model in our study.

Supplementary Material D. Accuracy Metric for Regression Task

A regression model entails predicting a numerical output, y, based on input data X. Evaluating the accuracy of such a model involves assessing how well the predicted output, y_{pred}, matches the true output, y_{true}. This section delves into the metrics employed in this research to gauge the accuracy of the model.

Supplementary Material D.1. R-squared (R2) Score

The R2 score is a statistical indicator used to assess the quality of fit of a regression model. It sheds light on how well the target variable's true values and the anticipated values of the model match each other. This is how the R^2 score is determined:

$$\mathrm{R}^2 = 1 - \frac{\sum_{i=1}^{n} (y_{true,i} - y_{pred,i})^2}{\sum_{i=1}^{n} (y_{true,i} - \bar{y}_{true})^2} \tag{D.1}$$

where y_pred, i represents the predicted value of the target variable for the i-th data point, y_ttrue, i represents the observed value of the target variable for the i-th data point, $\bar{y}_{true}$ is the mean of the true values of the target variable, and n is the number of data points.

The range of the R^2 score is between 0 and 1. A higher score denotes a better prediction accuracy, with 0 denoting that predictions are inaccurate, and 1 denoting a perfect fit. It's crucial to remember that a high score does not always indicate a causal connection or the absence of overfitting.

Supplementary Material D.2. Root-Mean-Squared Error (RMSE)

The RMSE is a statistic used to assess the predictive performance of a regression model by measuring the average size of the discrepancies between predicted and observed values. It indicates how well the projected values match the actual values. The RMSE is determined as follows:

$$\mathrm{RMSE} = \sqrt{\frac{\sum_{i=1}^{n} (y_{true,i} - y_{pred,i})^2}{n}} \tag{D.2}$$

The RMSE is provided in exactly the same units as the target variable, making it simpler to read and compare to the original data scale. A smaller RMSE suggests that the results predicted by the model are more accurate, implying a better match. However, like with any statistic, RMSE should be seen in context and in conjunction with other assessment metrics in order to have a thorough picture of the model's performance.

Supplementary Material D.3. Mean Absolute Error (MAE)

Another statistic used to assess the accuracy of a regression model's predictions is mean absolute error (MAE). It measures the difference between anticipated and true values in the same way that RMSE does, but instead of squaring the differences, it takes the absolute value of the discrepancies. Because it does not give more weight to higher mistakes, MAE is more resistant to outliers. The MAE is determined as follows:

$$\mathrm{MAE} = \frac{\sum_{i=1}^{n} |y_{true,i} - y_{pred,i}|}{n} \tag{D.3}$$

The MAE is expressed in the identical units as the target variable, making it straightforward to read and compare to the original data scale. A lower MAE suggests that the model's predictions are

more accurate. When compared to RMSE, which squares the errors, MAE is advantageous when we wish to punish huge mistakes less. MAE, like R2 score and RMSE, should be used in conjunction with other assessment metrics to provide a complete picture of the model's performance.

Supplementary Material D.4. Ratio of RMSE to MAE

As mentionied above, both RMSE and MAE have the same units as the original data. This section shows how these values are related. To make the equation more readable, the absolute value of the residual between the predicted and true value, e_i, is expressed as follows:

$$e_i = |y_{true,i} - y_{pred,i}|. \tag{D.4}$$

Therefore, Equations D.2 and D.3 can be modified by the followings:

$$\text{RMSE}^2 = \frac{\sum_{i=1}^{n} e_i^2}{n}, \tag{D.5}$$

$$\text{MAE}^2 = \frac{(\sum_{i=1}^{n} e_i)^2}{n^2}. \tag{D.6}$$

Let's consider the concept of variance in the context of a random variable X. Variance, denoted as Var(X), measures the extent to which individual data points in a dataset deviate from the mean of that dataset. It is a statistical measure of the dispersion or spread of the data points. The variance of a random variable X is calculated using the following formula:

$$\begin{aligned} \text{Var}(X) &= \text{E}(X^2) - (\text{E}(X))^2 \\ &= \frac{\sum_{i=1}^{n} x_i^2}{n} - \frac{(\sum_{i=1}^{n} x_i)^2}{n^2} \end{aligned} \tag{D.7}$$

where x_i represents the value of the random variable X for the i-th data point. If the variable X is replaced with the residual error, the equation can be arranged as following:

$$\text{RMSE}^2 - \text{MAE}^2 = \text{Var}(e). \tag{D.8}$$

Also, the e_i that only takes values greater than or equal to 0, mean value of e is equal to MAE, the ratio of RMSE to MAE can be expressed as

$$\frac{\text{RMSE}}{\text{MAE}} = \sqrt{1 + \frac{\text{Var}(e)}{\text{MEAN}(e)^2}}. \tag{D.9}$$

Let us assume that the error follows a normal distribution with mean 0 and standard deviation σ. The distribution of the absolute value of the error (=e_i) is then the distribution of the absolute value of the normal distribution. The probability density function, f, is defined as follows:

$$\text{f} = \frac{2}{\sqrt{2\pi}\sigma} \exp\left(-\frac{e^2}{2\sigma^2}\right). \tag{D.10}$$

Using the probability density function, the mean and variance can be computed as:

$$\begin{aligned}\mathrm{MEAN}(e) &= \int_0^{\infty} e \cdot \mathrm{f} de \\ &= \int_0^{\infty} e \cdot \frac{2}{\sqrt{2\pi}\sigma} \exp\left(-\frac{e^2}{2\sigma^2}\right) de \\ &= \sqrt{\frac{2}{\pi}}\sigma\end{aligned} \tag{D.11}$$

$$\begin{aligned}\mathrm{Var}(e) &= \int_0^{\infty} (e - \mathrm{MEAN}(e))^2 \cdot \mathrm{f} de \\ &= \int_0^{\infty} (e - \sqrt{\frac{2}{\pi}}\sigma)^2 \cdot \frac{2}{\sqrt{2\pi}\sigma} \exp\left(-\frac{e^2}{2\sigma^2}\right) de \\ &= \left(1 - \frac{2}{\pi}\right) \sigma^2\end{aligned} \tag{D.12}$$

Finally, the ratio of RMSE to MAE can be expressed by

$$\begin{aligned}\frac{\mathrm{RMSE}}{\mathrm{MAE}} &= \sqrt{1 + \frac{\left(1 - \frac{2}{\pi}\right)\sigma^2}{\left(\sqrt{\frac{2}{\pi}}\sigma\right)^2}} \\ &= \sqrt{\frac{\pi}{2}} \approx 1.253.\end{aligned} \tag{D.13}$$
